# Recyclable and eco-compatible polymer composite nanofibers for broad-spectrum dye remediation

**DOI:** 10.1039/d5lp00307e

**Published:** 2026-01-19

**Authors:** Kritika Rajput, Jesus Vazquez Chavez, Ana B. Jorge Sobrido, Manish Dev Sharma, Colin R. Crick

**Affiliations:** a School of Engineering and Materials Science, Queen Mary University of London Mile End Road E1 4NS London UK c.crick@qmul.ac.uk; b Department of Physics and Centre of Advanced Studies in Physics, Panjab University Sector-14 Chandigarh 160014 India

## Abstract

Photocatalytic nanoparticles (NPs) can be effective in degrading synthetic dyes, but their practical application is often limited by challenges in handling, recovery and recycling, leading to catalyst loss during use. In this study, firstly, CuO–ZrO_2_ hybrid nanoparticles were synthesised using a facile co-precipitation method at pH 7 and incorporated into the polyacrylonitrile (PAN) polymer at 20, 40, and 60 wt% loadings, followed by electrospinning and thermal stabilization (250 °C, 12 h) to obtain novel recyclable CuO–ZrO_2_@PAN composite nanofiber mats. To the best of our knowledge, this is the first report on the fabrication and application of electrospun PAN nanofibers incorporating CuO–ZrO_2_ hybrid nanoparticles. Among these, the 40 wt% CuO–ZrO_2_@PAN sample exhibited the highest photocatalytic performance. The optimized composite demonstrated broad-spectrum activity against structurally distinct azo and thiazine dyes (25 ppm), achieving >96% removal of Methylene Blue, ∼90% of Congo Red, ∼85% of Bismarck Brown, and ∼75% of Reactive Black within 60 minutes under irradiation. SEM-EDS analysis confirmed uniform nanoparticle immobilization within the fiber matrix, enabling facile recovery and reuse. Seed germination assays on *Vigna radiata* seeds further validated the eco-compatibility of the synthesised nanofibers, indicating no phytotoxicity effects up to 75 ppm. These results identify CuO–ZrO_2_@PAN nanofibers as a durable, recyclable and environmentally safe photoactive filter system for dye contaminated wastewater treatment.

## Abstract 2

Photocatalytic nanoparticles (NPs) can be effective in degrading synthetic dyes, but their practical application is often limited by challenges in handling, recovery and recycling, leading to catalyst loss during use.

## Graphical abstract

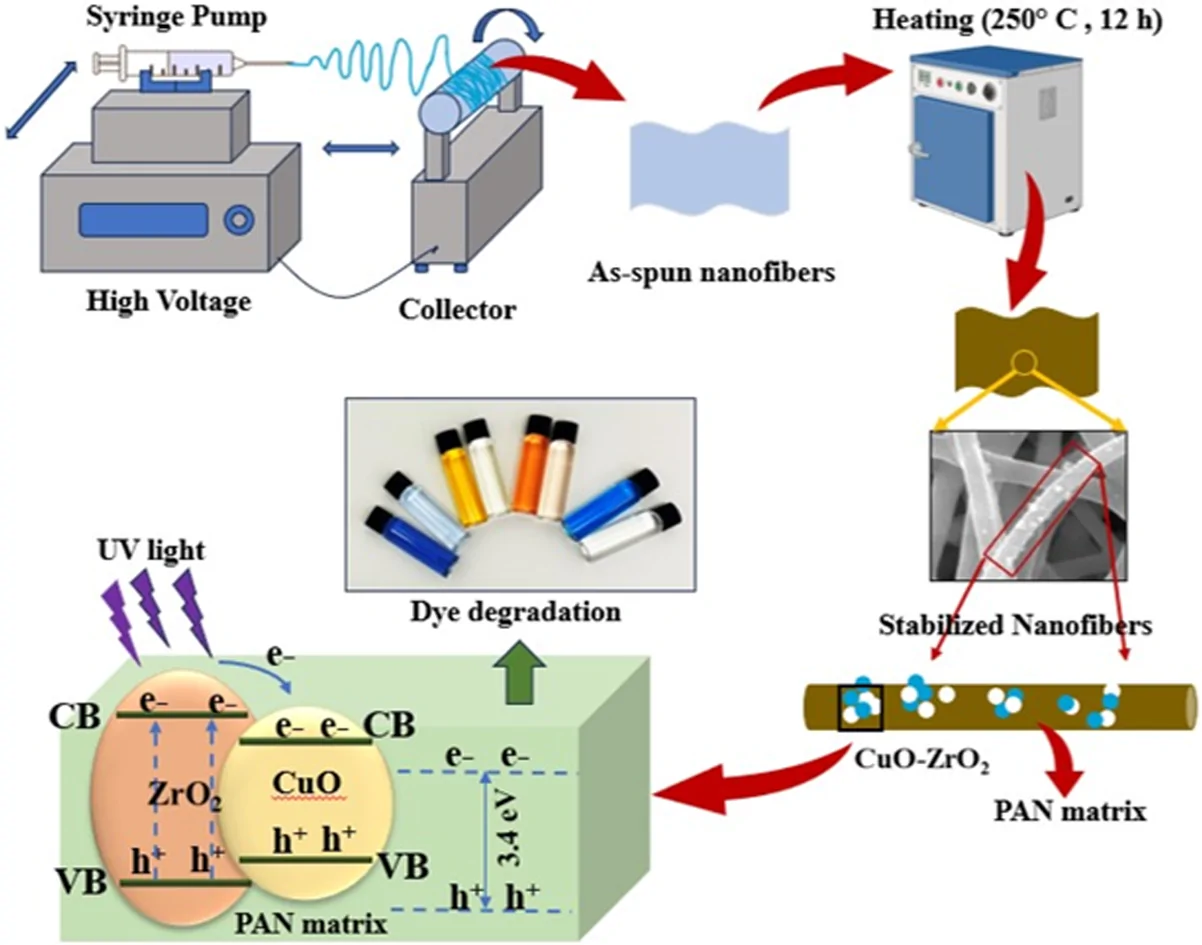

## 1. Introduction

The increasing demand for synthetic dyes in industries such as textiles, plastics, and cosmetics has led to the release of significant quantities of these persistent pollutants into aquatic environments.^1,2^ These dyes such as Methylene Blue (MB), Congo Red (CR), Reactive Black (RB), Bismarck Brown (BB), *etc*. are known for high chemical stability and resistance to biodegradation or photolysis alone due to their complex aromatic and azo structures.^3,4^ Consequently, heterogeneous catalytic and photocatalytic degradation approaches employing metal oxide based nanoparticles have gained wide attention.^5–8^ Nanoparticles (NPs) have exhibited superior efficacy in synthetic dye removal owing to their large surface area, tenable surface chemistry, and enhanced reactivity, which enables the adsorption and degradation of a wide range of contaminants.^9,10^ Metal oxide nanoparticles have demonstrated strong catalytic and photocatalytic properties in wastewater treatment applications, and metal oxide nanoparticles such as TiO_2_, ZnO, *etc*. have been extensively studied for their desired properties.^11–13^

To enhance the functional efficiency of many wide band gap metal oxide nanoparticles such as ZrO_2_, TiO_2_, *etc*., extensive research has been focused on the rational design of metal oxide-based nanocomposites (NCs) with tailored physicochemical properties. The integration of metal oxides within composite systems enables modulation of parameters such as surface charge, crystallinity, defect concentration and electronic structure, which directly influence the adsorption and catalytic performance. Diverse approaches (physical, chemical and biological) are used for the synthesis of novel NCs.

Among these, chemical methods such as sol–gel synthesis, hydrothermal processes, thermal decomposition and co-precipitation remain widely used owing to their controllability over nucleation and growth kinetics, low cost and environmental friendliness.^14^ For example, Rajput *et al.* prepared ZrO_2_ NPs *via* a hydrothermal method and obtained a band gap of ∼4.58 eV and synthesised CuO–ZrO_2_ NCs with a decreased band gap of 2.4 eV *via* a co-precipitation method.^15,16^ Kubiak *et al.*, using the hydrothermal route, prepared ZnO/TiO_2_, ZrO_2_/TiO_2_ and MoS_2_/TiO_2_ NCs, which exhibited enhanced antibacterial and photocatalytic activities compared to pristine TiO_2_.^17^

However, the direct application of these NCs and NPs in aqueous media presents several limitations, including particle agglomeration, difficulty in recovery, and potential secondary pollution due to nanoparticle leaching into water bodies as toxins.^18,19^ These limitations hinder reusability and raise concerns regarding secondary contamination, indicating the need for catalyst systems that combine high activity with structural stability, environmental safety and applicability on a broader class of dyes. To address these challenges, immobilization of NPs within a solid support matrix to form a filter sheet and functionalization of nanofibers have been explored as a means to enhance stability, improve handling, and facilitate reusability of such membranes.^20,21^ The electrospun polymeric nanofibers (NFs), in particular, have gained prominence as promising host matrices for NP immobilization due to their high surface-to-volume ratio, interconnected porosity and structural flexibility as reported.^22,23^

Amongst polymers, the semicrystalline organic polymer polyacrylonitrile (PAN) is widely used in electrospinning. It is characterized by repeated nitrile (–CN) groups along its carbon backbone. The –CN group tends to exhibit strong dipole–dipole interactions due to electron-rich nitrogen and electron-deficient carbon atoms, enabling PAN to act as a hydrogen bond acceptor. These polar interactions play a crucial role in enabling surface functionalization and chemical grafting and strong affinity for metal oxides, making PAN a versatile platform for membrane fabrication.^24^ Furthermore, the strong intermolecular bonding between PAN chains imparts significant mechanical robustness and resistance to many organic solvents.

As a result, PAN fibers are widely employed in applications requiring structural durability and chemical stability including pervaporation and organic solvent filtration membranes particularly through electrospinning.^24^ This offers an opportunity to design hybrid nanofiber systems in which the polymer not only stabilizes the nanoparticles but also actively modulates interfacial charge transfer in the fiber matrix. For example, Badsha *et al.* fabricated PAN-CMQD electrospun nanofiber membranes by laser ablating and bending curcumin quantum dots (CMQD) and PAN for mitigation of heavy metal contaminants such as Pb^2+^, Cd^2+^, Mn^2+^, Fe^2+^ and Cr^2+^ along with MB dye degradation.^25^ ZnO/PPy/PAN nanofibers were studied by Yan *et al.*, which exhibited good recyclability and maintained a removal efficiency of 79% for RhB after 5 consecutive cycles. The enhanced photocatalytic efficiency was ascribed to the synergic effect of n–p heterojunction catalysis and PAN nanofiber adsorption, resulting in a narrower band gap and increased charge separation.^26^

The presented work explores the fabrication of a novel CuO–ZrO_2_@PAN nanofiber composite membrane *via* co-precipitation and electrospinning processes at variable CuO–ZrO_2_ NP dosages (20, 40 and 60 wt%) in the PAN matrix. Their efficiency in the catalytic degradation of Methylene Blue (MB) dye under ambient conditions was systematically evaluated. The samples were characterized by techniques such as XRD, FTIR, TGA, SEM-EDS, UV-DRS and HR-TEM to envisage the enhanced physicochemical properties. The optimized nanofiber composite was also tested for its ability to successfully degrade other structurally distinct azo dyes (Congo Red (CR), Bismarck Brown (BB) and Reactive Black (RB)), to demonstrate its versatility and potential for broad-spectrum dye remediation in the future. Finally, an ecotoxicity analysis of the CuO–ZrO_2_@PAN nanofibers (NFs) was done at different fiber dosages on *Vigna radiata* (green gram) seeds to study their environmental impact. Hence, this work not only provides a scalable route for fabricating novel CuO–ZrO_2_@PAN nanofibers *via* a facile synthesis method but also advances the development of hybrid catalysts for efficient, reusable and ecofriendly broad spectrum dye remediation.

## 2. Experimental

### 2.1. Chemical reagents

All reagents – copper(ii) chloride dihydrate (CuCl_2_·2H_2_O), zirconium oxychloride (ZrOCl_2_·8H_2_O) (purity, 99.98%), ammonium hydroxide solution (NH_4_OH) (28.0–30.0% NH_3_ basis), hexadecyltrimethylammonium bromide (CTAB) (purity, >98%), and Methylene Blue, Reactive Black 5, Bismarck Brown Y, and Congo Red (purity, >97.0%) dyes – were purchased from Sigma-Aldrich (India). Polyacrylonitrile (PAN) (M.W. 150 000) and *N*,*N*-dimethylformamide (DMF) were purchased from Thermo Fisher Scientific (UK). No further purification of the reagents was carried out, and they were used as purchased.

### 2.2. CuO–ZrO_2_ nanocomposite synthesis

Typically, 10 mg of CTAB was dissolved in 20 ml of distilled water under magnetic stirring for 20 min; then the precursors – 500 mg each of ZrOCl_2_·8H_2_O and CuCl_2_·2H_2_O – were dissolved under continuous stirring and kept overnight at 40 °C. As the initial pH of the metal salt solution was 1.6, ammonium hydroxide was introduced dropwise until a pH value of 7 was reached. A blue precipitate was formed once the pH was adjusted. This precipitate was then separated using a centrifuge (Eppendorf Microcentrifuge 5425) operating at 10 000 rpm for 15 min. The obtained blue slurry was thoroughly washed with distilled water followed by ethanol in order to remove all the unreacted species. The slurry was then dried in an oven for 2 h at 50 °C, and the resultant blue powder was further calcined for 2 h at 600 °C to remove any impurities and to get better phase purity and crystallinity.^16,27–29^

### 2.3. Fabrication of photo-active CuO–ZrO_2_@PAN nanofibers

The prepared CuO–ZrO_2_ nanoparticles were added at 20, 40, and 60 wt% dosages to 0.524 g of PAN in 5 ml of *N*,*N*-dimethylformamide (DMF) respectively and stirred for 12 h at room temperature. As an example, a 20 wt% sample contains 0.104 g of CuO–ZrO_2_ NPs and 0.524 g of PAN in 5 ml of DMF. The CuO–ZrO_2_@PAN NFs were then deposited *via* electrospinning on an aluminium rotating drum collector set up at 80 rpm to prevent alignment while obtaining a homogeneous thickness on a moving syringe pump platform by electro-spinning at 15 kV, employing a blunt 21G needle, considering a 15 cm tip-to-collector distance, and a 0.7 ml h^−1^ pumping rate, followed by stabilisation at 250 °C for 12 h in a hot air oven.

### 2.4. Characterization

The evaluation of the fabricated CuO–ZrO_2_@PAN NFs involved several characterization techniques. Firstly, X-ray diffraction (XRD) analysis was performed in reflection mode utilizing a PANalytical X'Pert PRO MPD, including a high-throughput screening XYZ stage, an X-ray focusing mirror, and a PIXcel detector. Cu Kα radiation was used to record diffraction data over a 5–120° 2*θ* range. Peak identification was performed using the JAD program.

Thermogravimetric analysis (TGA) was conducted under an inert atmosphere using a TA Instruments TGA 5500. The sample was heated from room temperature to 800 °C at a rate of 10 °C min^−1^. Attenuated total reflectance–Fourier transform infrared (ATR-FTIR) spectra were recorded using a Bruker Tensor 27 spectrometer within a wavenumber range of 500–4000 cm^−1^. Scanning Electron Microscopy (SEM) imaging and Energy-Dispersive X-ray Spectroscopy (EDS) were performed using an FEI Inspect F system operating at an acceleration voltage of 10–20 kV. To improve electrical conductivity during SEM analysis, samples were sputter-coated with a thin gold layer using an automatic sputter coater. Transmission electron micrographs were recorded on a JEOL JEM-F200, a high-resolution scanning transmission electron microscope (HR-TEM). The sizes of the particles and the fibers formed were calculated to be ∼9.71 nm and 291 nm, respectively, using ImageJ software, in addition to examination of the XRD plots using the Scherrer equation.^30^ The band gap was determined by Diffuse Reflectance Spectroscopy (DRS) to be ∼3.4 eV using the Tauc-plot, following the relation given in eqn (1).^31^

1 (*αhν*)^1/*n*^ = *A*[*hν* − *E*_g_]

In this equation, *α* is the absorbance coefficient, *h* is Planck's constant, *ν* is the frequency, and *E*_g_ is the band gap. To evaluate the progress of the reactions, a UV–visible spectrophotometer (PerkinElmer Lambda 35 UV-Vis spectrometer) in the range of 300–700 nm was employed.

#### 2.4.1. Photocatalytic removal of Methylene Blue dye

The stock solution of the cationic MB dye was prepared by dissolving the dye in double-distilled water to achieve a concentration of 50 mg L^−1^. Working solutions were prepared by diluting the stock solution with double-distilled water. The photocatalytic efficiency of the CuO–ZrO_2_@PAN NFs was then assessed by monitoring the reduction of dye concentration under various experimental conditions including only exposure to UV light irradiation (Fig. S1). The removal process was conducted under UV-C light irradiation using a 6 W lamp (Cole-Parmer Handheld UV Lamp) of wavelength 254 nm, inside an insulated photoreactor, positioned 5 cm above the solution surface with a light intensity of approximately 3.1 mW cm^−2^.

The reaction solution was contained in a borosilicate glass beaker (50 ml) with an effective working volume of 20 ml. The beaker was placed centrally beneath the lamp to ensure uniform illumination with continuous magnetic stirring at 600 rpm and EEO = 158.7 kWh per m^3^ per order. During the experiment, a measured amount of the prepared photocatalyst was added to the dye solutions, which were continuously stirred magnetically for a specified period. The mixture was first kept in the dark for 30 min and then irradiated with UV-C light for 1 h. Subsequently, at selected time intervals, 2 mL aliquots were taken and filtered through a 0.22 μm PTFE membrane to determine the residual MB dye concentration in solution. Quantification was performed using a PerkinElmer Lambda 35 UV-vis spectrometer. The photocatalytic degradation efficiency was calculated using eqn (2), where *C*_0_ denotes the initial dye concentration and *C* represents the concentration after photocatalytic treatment.^15^ The degradation kinetics were analysed by fitting the data to isotherm models along with pseudo-first-order and second order kinetic models.2
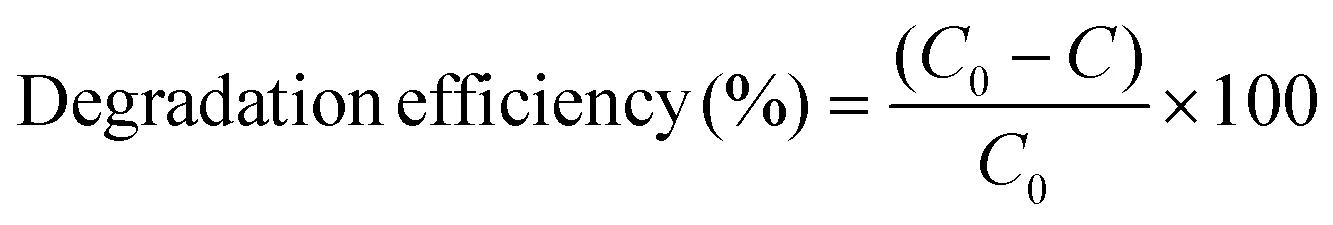


Additionally, a radical trapping experiment was conducted using 2 mM isopropyl alcohol (IPA), 2 mM ascorbic acid (ASC), 2 mM potassium dichromate (K_2_Cr_2_O_7_), and 2 mM ethylenediaminetetraacetate (EDTA) as scavengers. These agents were employed to selectively capture reactive species, including hydroxyl radicals (˙OH), superoxide radicals (O_2_^−^), electrons (e^−^), and photogenerated holes (h^+^), respectively.^15^

#### 2.4.2. Ecotoxicological assay

The phytotoxicity of CuO–ZrO_2_@PAN nanofibers was primarily assessed using a seed germination assay, with root length serving as the primary evaluation parameter. *Vigna radiata* (green gram) was selected for toxicity analysis.^32^ The seeds were pre-soaked in deionized water overnight and subsequently incubated in a temperature-controlled chamber set at 37 °C. For each test condition, 10 viable seeds were exposed to 10 mL of CuO–ZrO_2_@PAN nanofiber solutions at varying concentrations (0 (control), 25, 50, 75, and 100 ppm) in individual glass Petri dishes. To simulate natural soil darkness, the entire setup was kept in a light-excluded environment, while the incubator temperature was maintained at 37 °C to promote seed sprouting. After 72 hours of incubation, the rootlets were counted, and their lengths were recorded to analyse the toxicity of nanofibers on plant growth.

## 3. Results and discussion

### 3.1. Morphological and chemical analysis

**Fig. 1:**
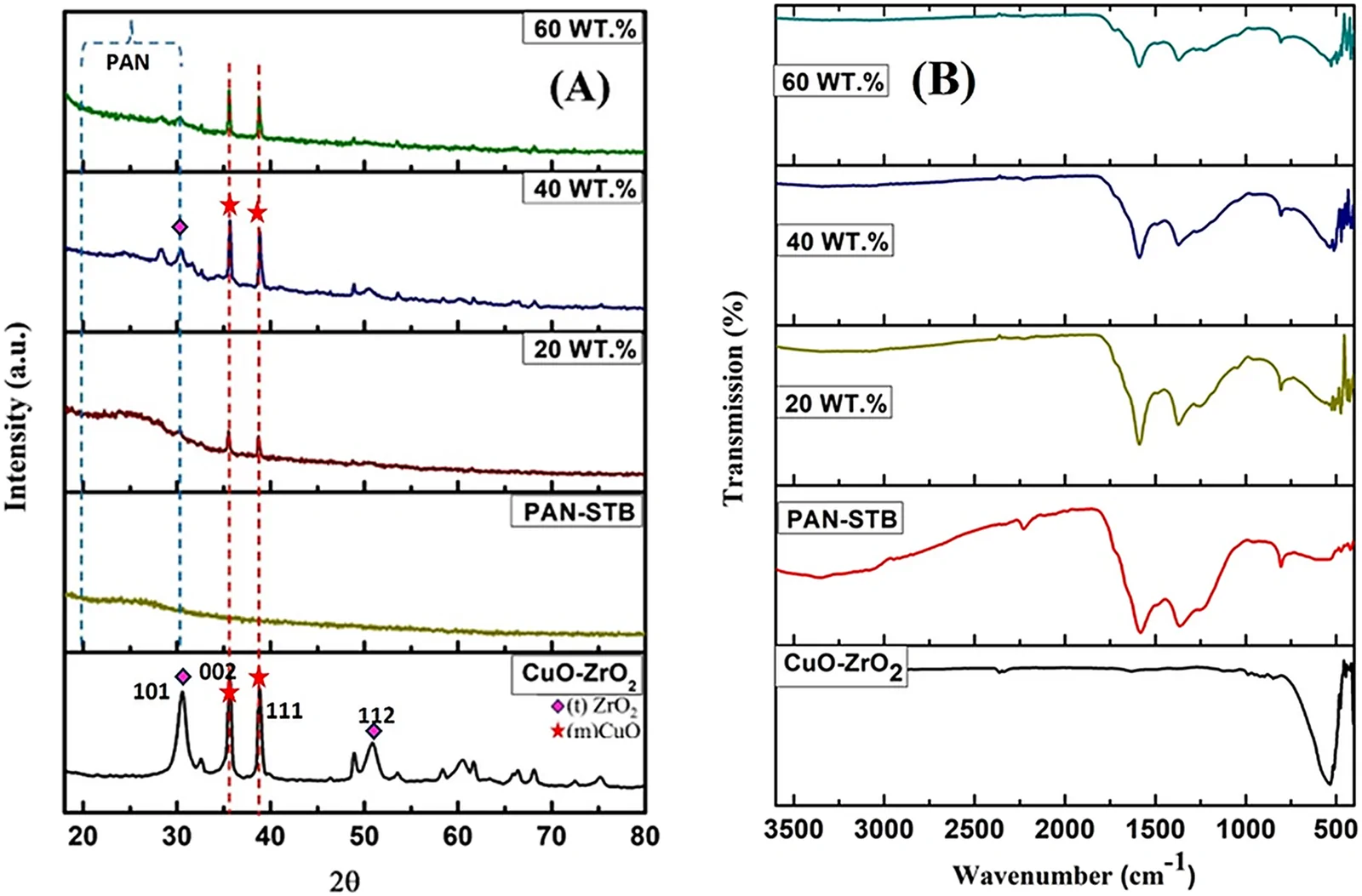
(A) XRD and (B) FTIR analyses of all the compositions along with pure PAN and pure CuO–ZrO_2_ NPs. All FTIR plots are plotted over the same transmission range (*y*-axis; 0–100% transmission).

Fig. 1(A) displays the XRD patterns of CuO–ZrO_2_ NPs, pure PAN stabilized nanofibers and CuO–ZrO_2_@PAN nanofibers with varying CuO–ZrO_2_ loadings (20, 40 and 60 wt%). The pattern of CuO–ZrO_2_ shows distinct and sharp peaks in the 2*θ* range of 20°–80°, corresponding to CuO and ZrO_2_. The major reflections around 2*θ* = 28.2°, 31.5°, 35.5° and 38.3° are attributed to the monoclinic CuO (tenorite phase, JCPDS no. 48-1548) and tetragonal or monoclinic ZrO_2_ (JCPDS no. 37-1484 or 17-0923) corresponding to the (111), (002) and (101) planes, respectively, confirming a well-crystallised mixed oxide system.^33^

In contrast, due to the amorphous polymeric nature, the pure PAN stabilised (PAN-STB) fibers show a broad hump in the region of 20°–30° (Fig. 1(A)).^34^ The incorporation of the CuO–ZrO_2_ NPs into the PAN-STB matrix results in the appearance of crystalline peaks corresponding to the oxide phases, which become more prominent with increasing oxide loading. The 20 wt% composite shows weak but noticeable peaks, while the 40 wt% sample exhibits a significantly enhanced crystalline phase, reflected in higher peak intensities. Interestingly, at 60 wt%, the peak intensities do not increase further and appear slightly reduced compared to 40 wt%. This counterintuitive behaviour can be attributed to factors like poor dispersion of NPs within the matrix due to particle agglomeration^35^ or high oxide loading in a fixed solvent volume, leading to sedimentation or non-uniform distribution, affecting the local crystallite density and hence the diffracted signals.^36^

To investigate the chemical interactions and structural evolution during the formation of CuO–ZrO_2_@PAN nanofibers, FTIR analysis of the nanomaterial with varying CuO–ZrO_2_ loadings (20, 40 and 60 wt%) was performed (Fig. 1(B)). The characteristic peaks observed in all spectra confirm the presence of PAN functional groups as well as integration of metal oxide components. Notably, the typical adsorption band (present in the PAN-STB matrix) at 2243 cm^−1^, corresponding to the stretching vibration of the nitrile (–C≡N) group,^37,38^ was absent or was significantly suppressed in all the loaded samples. This observation strongly suggests that the nitrile group in PAN underwent cyclization reactions during the stabilisation process, potentially leading to the formation of conjugate or aromatic ladder-like structures.^37^ The presence of CuO–ZrO_2_ might have facilitated these reactions by acting as a catalyst or through coordination with the nitrile groups.

Further evidence of structural changes is seen in the emergence and enhancement of peaks around 1600 cm^−1^, attributed to the C=N stretching, indicating the development of conjugated systems as a result of PAN stabilisation.^38,39^ This is also supported by the disappearance of the nitrile bond mentioned above. In addition, new or intensified bands in the 500–700 cm^−1^ region can be associated with metal oxygen vibrations, particularly Cu–O and Zr–O bonds.^16,33^ This confirms the successful incorporation of CuO–ZrO_2_ NPs into the PAN matrix. The shift or broadening of the band around 3400 cm^−1^ of O–H/N–H bands further support the chemical interactions and structural rearrangements during the composite formation.^38^ These changes indicate strong interfacial interactions between the polymer matrix and the CuO–ZrO_2_ NPs. The TGA technique was employed to evaluate the thermal stability and decomposition profiling. The graph showed a multi-step weight loss pattern which was divided into different sections: (i) initial weight loss up to ∼150 °C which was attributed to the removal of volatile solvents and water molecules, (ii) the second stage (150–500 °C) of PAN stabilization and decomposition and lastly (iii) the third stage (>600 °C) was attributed to the carbonization and decomposition of organic matter and the remaining weight % was attributed to the residual metal oxide particles (Fig. S2).

**Fig. 2:**
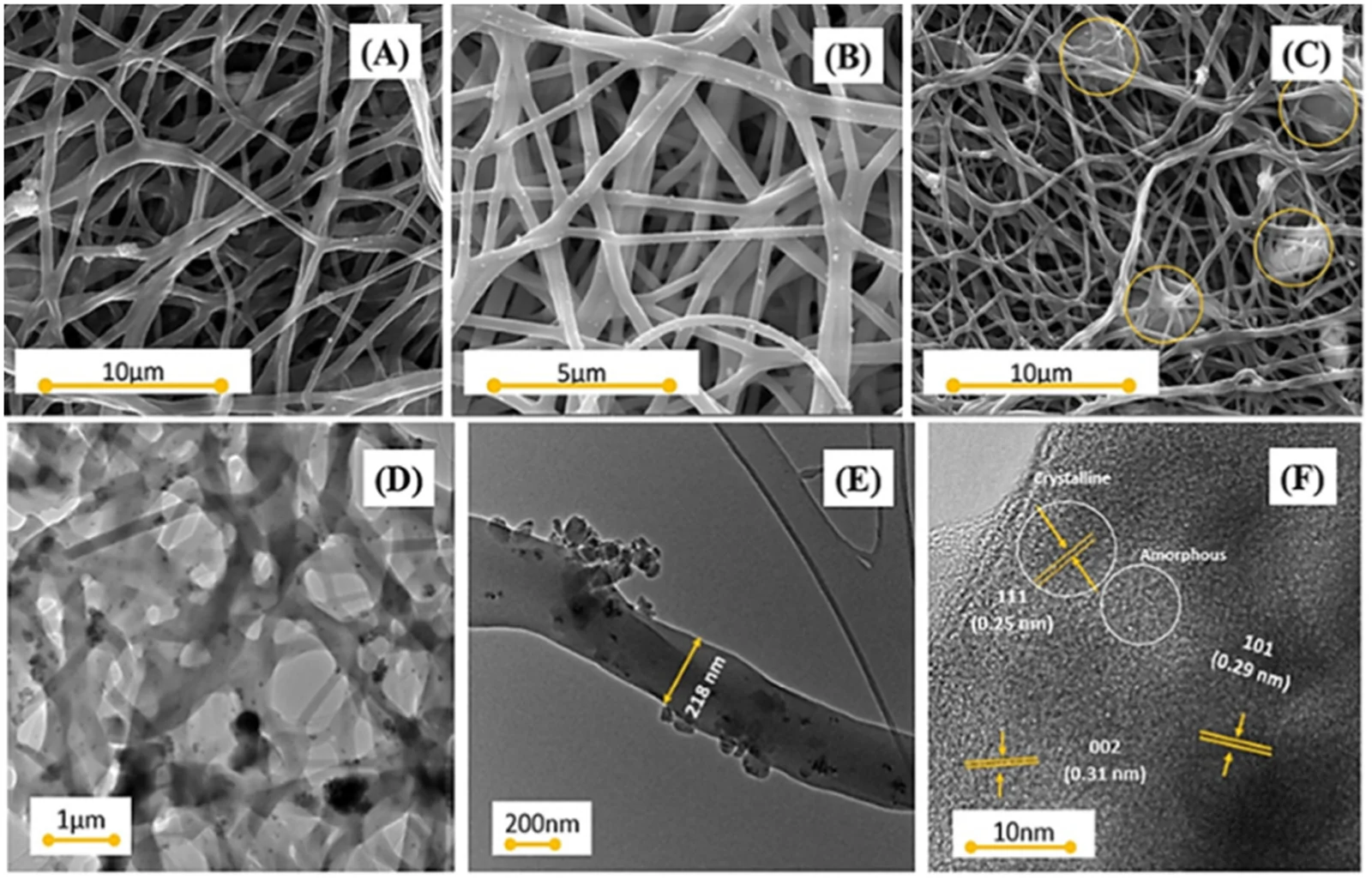
SEM images of (A) 20 wt%, (B) 40 wt%, and (C) 60 wt% samples. (D) TEM images of the 40 wt% sample, (E) the size of the fiber and (F) different crystalline planes of CuO–ZrO_2_ NPs in the amorphous PAN matrix.

The SEM analysis of the CuO–ZrO_2_@PAN NFs revealed significant morphological differences influenced by the CuO–ZrO_2_ content (Fig. 2(A–C)). At 20 wt% loading, nanoparticles did not appear well distributed on the surface of the fibers, maintaining a relatively smooth surface of just PAN fibers, while 40 wt% loading showed uniform distribution of NPs on the fiber surface, hence increasing roughness, potentially enhancing the surface area and catalytic efficiency. However, at 60 wt% loading, nanoparticle agglomeration was observed to be pronounced, reducing uniformity and leading to potential mechanical instability.^40^

The literature suggests that low to moderate nanoparticle loadings (∼10–40 wt%) favour optimal fiber formation, whereas high loadings (>50 wt%) induce defects and reduce structural integrity.^41^ The surface roughness observed in both 40 and 60 wt% loadings (Fig. S3) may enhance photocatalytic efficacy by increasing active sites, but the excessive aggregation in 60 wt% fibers could reduce the available surface area for catalytic interaction due to saturation. Thus, 40 wt% CuO–ZrO_2_ NP loading appears optimal, ensuring both structural stability and enhanced surface properties, whereas 60 wt% fibers compromise uniformity, dispersion and available active sites, which could negatively impact the mechanical and photocatalytic performance.

**Fig. 3:**
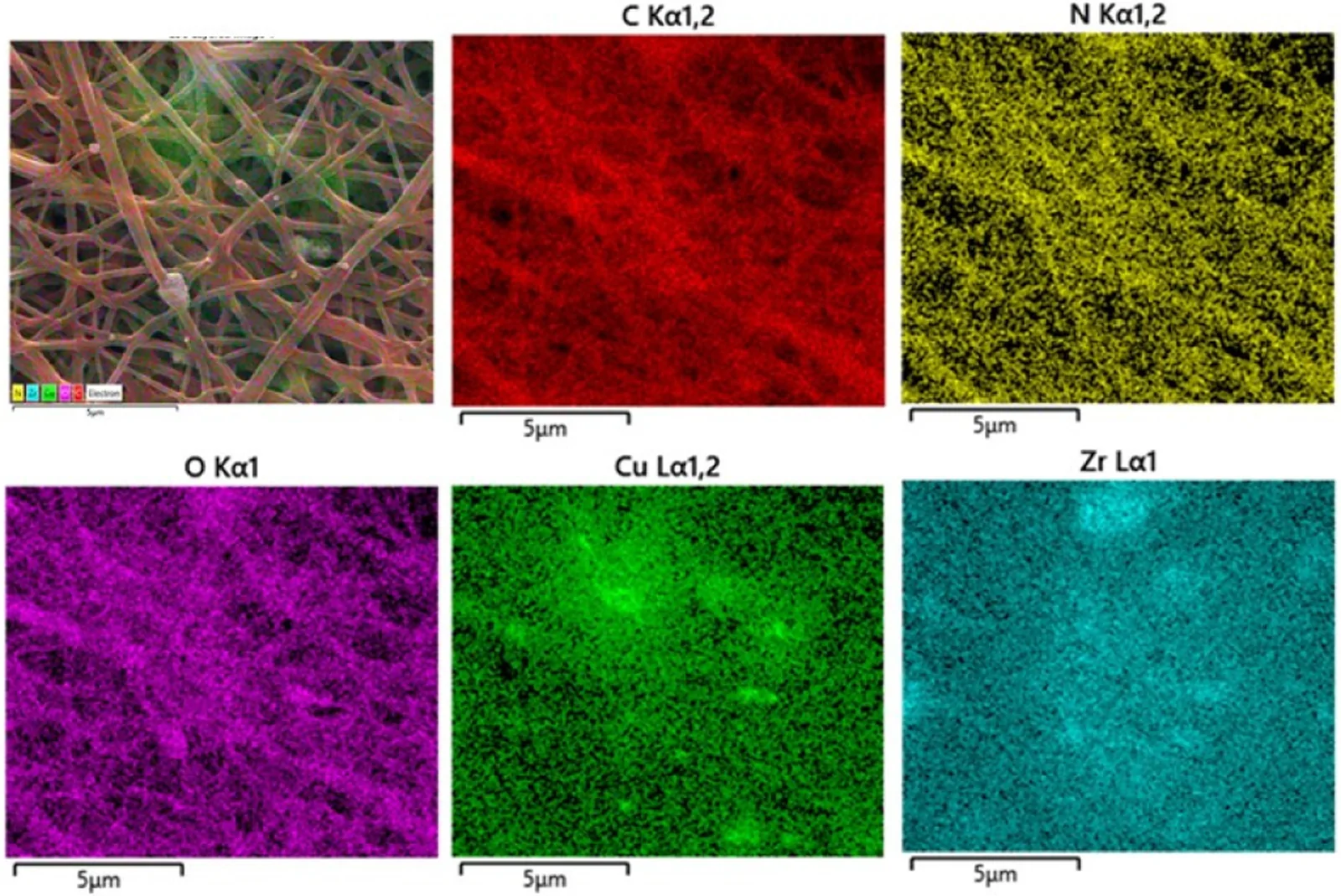
Elemental mapping of the composition of the optimized CuO–ZrO_2_@PAN fibers.

These findings along with the experimental results of the removal of MB dye suggested that the 40 wt% loading was the optimal concentration of nanoparticles for the formation of the fibers; hence, TEM analysis of 40 wt% CuO–ZrO_2_@PAN nanofibers was carried out (Fig. 2(D)–(F)). TEM images revealed a well-defined porous nanofibrous network with an average fiber diameter of ∼291 nm (Fig. S4(A)), ensuring a high surface area beneficial for adsorption and catalytic applications. The dark contrast regions in the images confirm the uniform dispersion of CuO–ZrO_2_ nanoparticles along the nanofiber surface, with nanoparticles measuring in the range of 10–30 nm in diameter (Fig. S4(C and D)). HR-TEM images further validated the crystalline nature of the nanocomposites, with distinct lattice fringes corresponding to the (111) and (002) planes of monoclinic CuO (*d* = 0.25 nm, 0.31 nm) and the (101) plane of tetragonal ZrO_2_ (*d* = 0.29 nm), confirming the formation of CuO–ZrO_2_ heterostructures (Fig. 2(F)).^42^ These findings align well with XRD analysis, which confirms the presence of mixed-phase CuO–ZrO_2_ nanocrystals. The EDS spectra (Table 1) and elemental mapping (Fig. 3) of the 40 wt% fibers were also recorded for compositional studies.

**Table 1:** EDS spectral result for the elemental analysis of the optimized NFs

Elements	Elemental analysis (wt%)
C	56.4
N	20.8
O	16.1
Cu	3.6
Zr	3.0

The well-defined crystallinity and nanoscale dispersion of these metal oxide NPs in the fiber matrix are expected to enhance their surface reactivity and catalytic efficiency. Furthermore, to analyse the band gap energy of the formed CuO–ZrO_2_@PAN NFs, diffuse reflectance spectroscopy (DRS) was used, and the band gap was determined to be ∼3.4 eV using the Tauc-plot (Fig. S4(B)). The spectrum showed broad absorbance in the wavelength range of 200–700 nm (Fig. S4(B) (inset)), similar to a study conducted by Hartati *et al.*,^43^ indicating efficient light harvesting ability, suggesting the presence of multiple electronic transitions. The absorption edge lying around 280–300 nm indicates that the NFs have a potential photocatalytic characteristic in the UV region. Hence, UV-C (254 nm) irradiation was used for all the discussed studies below.

### 3.2. MB dye removal studies

#### 3.2.1. Effect of the pH of dye solution

**Fig. 4:**
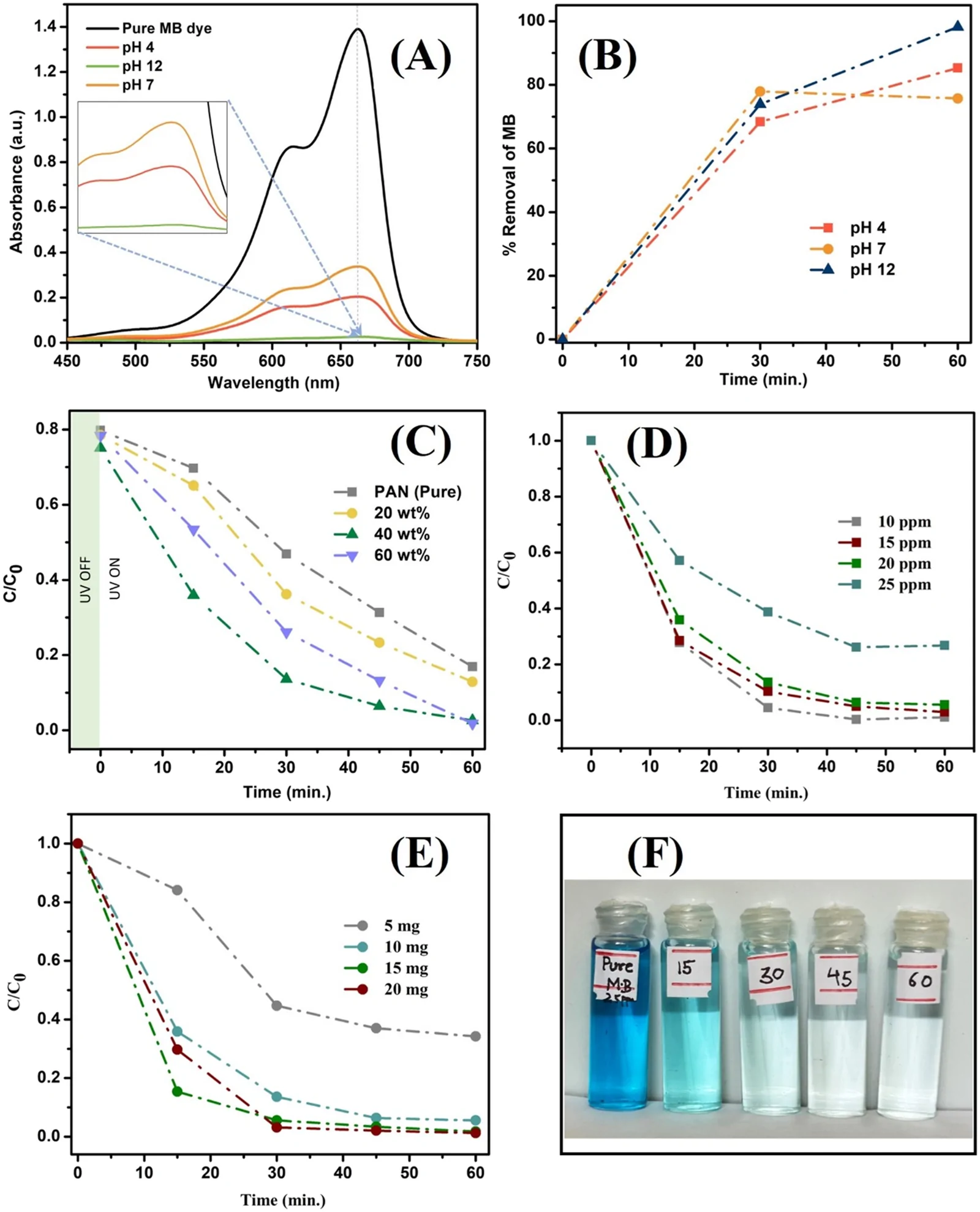
Comparative analysis of MB dye removal at (A, B) different pH values (4, 7, and 12), (C) variable catalyst composition (0–60 wt%), (D) different dye concentrations (10–25 ppm) and (F) visual results under optimal conditions (*C*_0_ = 20 ppm, 0.02 g ml^−1^ of 40 wt% CuO–ZrO_2_@PAN NFs at RT).

The effect of the pH of the MB dye solution on the removal efficiency of the NFs was tested at pH 4, 7 and 12 (Fig. 4(A and B)). The maximum removal efficiency of ∼98% was observed at pH 12. The enhanced degradation at alkaline pH is attributed to the increased formation of hydroxyl radicals (˙OH), indicating that this plays a crucial role in oxidative dye breakdown.^44^ At pH 4, despite the presence of excess H^+^ ions that can scavenge hydroxyl radicals, the degradation efficiency remained high at approximately 90%, possibly due to enhanced catalytic surface charge interactions favouring dye adsorption.^45^ However, at neutral pH 7, the efficiency dropped to around 70%, likely due to a balance between hydroxyl radical formation and recombination effects reducing overall degradation. These findings indicate that an alkaline environment significantly enhances the photocatalytic performance of the catalyst for MB degradation.

#### 3.2.2. Effect of composition %

The effect of nanoparticle loadings (in wt.%) on the photocatalytic removal of MB dye was analysed over 60 minutes, as illustrated in Fig. 4(C). The results indicated that increasing the CuO–ZrO_2_ content significantly enhanced dye degradation under UV irradiation, with the pure PAN samples (*i.e.* already stabilised PAN) exhibiting the lowest removal efficiency. Interestingly, the 60 wt% sample also showed no significant degradation improvement compared to the 40 wt% sample, suggesting possible agglomeration due to higher nanoparticle loading, as observed in SEM, which may reduce active surface sites,^46,47^ which was also highlighted in the SEM results. The drastic reduction in *C*/*C*_0_ values under UV ON conditions, after keeping the system in the dark for 30 min in order to achieve adsorption equilibrium (Fig. S5(A)), confirms that photocatalysis plays the primary role in MB dye degradation. These findings underlined the critical role of nanofiber composition in optimizing photocatalytic efficiency, and hence, the 40 wt% sample under UV irradiation for 60 minutes was used as the optimal condition for all further studies.

#### 3.2.3. Effect of initial dye concentration

The removal trend of MB using CuO–ZrO_2_@PAN nanofibers demonstrates a strong dependence on the initial dye concentration (Fig. 4(D)). At lower concentrations (10 ppm and 15 ppm), rapid adsorption occurs within the first 30 minutes, leading to near-complete removal by 60 minutes (*C*/*C*_0_ = 0.087 approx.), suggesting an abundance of available active sites for dye molecules. However, at higher concentrations (20 ppm and 25 ppm), the removal efficiency initially slows, with 25 ppm showing the least reduction in *C*/*C*_0_ values over time, likely due to site saturation and increased dye–dye repulsion effects.^48^ Interestingly, 20 ppm exhibits the best removal efficiency by the end of 60 minutes (*C*/*C*_0_ = 0.016), indicating an optimal balance between dye molecule availability and nanofiber capacity. Beyond this point, excess dye may shield active sites, limiting further degradation drastically.^49^ The observed trend aligns with Langmuir adsorption behaviour (detailed in section 3.3), where the removal efficiency improves up to an optimal concentration before declining due to surface saturation.^50^ Additionally, at very high MB dye concentrations, limited diffusion and steric hindrance may restrict MB dye molecules from reaching deeper adsorption sites.^48,51^ This suggests that the prepared CuO–ZrO_2_@PAN nanofibers followed a monolayer adsorption mechanism, with catalytic and electrostatic interactions playing a crucial role in dye removal.

#### 3.2.4. Effect of catalytic dosage

Fig. 4(E) depicts the photocatalytic degradation profile of MB dye (20 ppm) over 60 minutes, represented as a normalised concentration ratio *C*/*C*_0_, for varying dosages (5 mg, 10 mg, 15 mg and 20 mg) of the synthesised CuO–ZrO_2_@PAN nanofibers. It was evident from the graph that an increase in catalyst dosage enhances the degradation efficiency, particularly within the first 30 minutes of UV irradiation. At a catalyst dosage of 5 mg, the degradation proceeds slowly, with only ∼60% removal of MB dye by 30 minutes (*C*/*C*_0_ ∼ 0.4), and a final value of ∼0.3 after 60 minutes. This suggested that the number of active sites available on the photocatalyst surface is insufficient to generate a substantial quantity of reactive oxygen species (ROS),^52^ primarily hydroxyl radicals (˙OH), which are responsible for oxidative degradation. Increasing the dosage to 10 mg resulted in a faster/more efficient degradation, achieving ∼85% reduction within the first 30 minutes and further decline beyond that. The trend continues with 15 mg and 20 mg dosages, where degradation exceeds 95% within 20–30 minutes and reaches 98% at the end of 60 minutes. This behaviour indicates an accelerated photocatalytic reaction, attributed to the higher density of active sites and increased adsorption of MB dye molecules on the catalyst surface.^51^ However, both 15 mg and 20 mg dosages exhibit similar performance; the incremental improvement from 15 mg to 20 mg by the end of 60 minutes is negligible. The plateauing effect around 50–60 minutes has been widely reported^51,52^ and is generally attributed to material agglomeration, increased light scattering that hinders the effective penetration of photons, as a result, reducing the amount of photogenerated electron–hole pairs.^52^ This suggests that the photocatalytic system reaches a saturation point beyond which additional catalyst does not significantly contribute to the degradation of the MB dye. The final pictorial degradation results are also shown in Fig. 4(F), under all the obtained optimal conditions from the above experiments, *i.e.* 20 mg of CuO–ZrO_2_@PAN with 40 wt%, at a dye solution pH of 12, for 20 ppm of MB dye in 60 minutes. Recyclability of the prepared photoactive filter/nanofibers was also studied for five consecutive cycles, observing a degradation capacity above 90% for almost the first 3 cycles and about 80% for the remaining cycles (Fig. S5(B)).

### 3.3. Kinetic studies and trapping experiments for active species

**Fig. 5:**
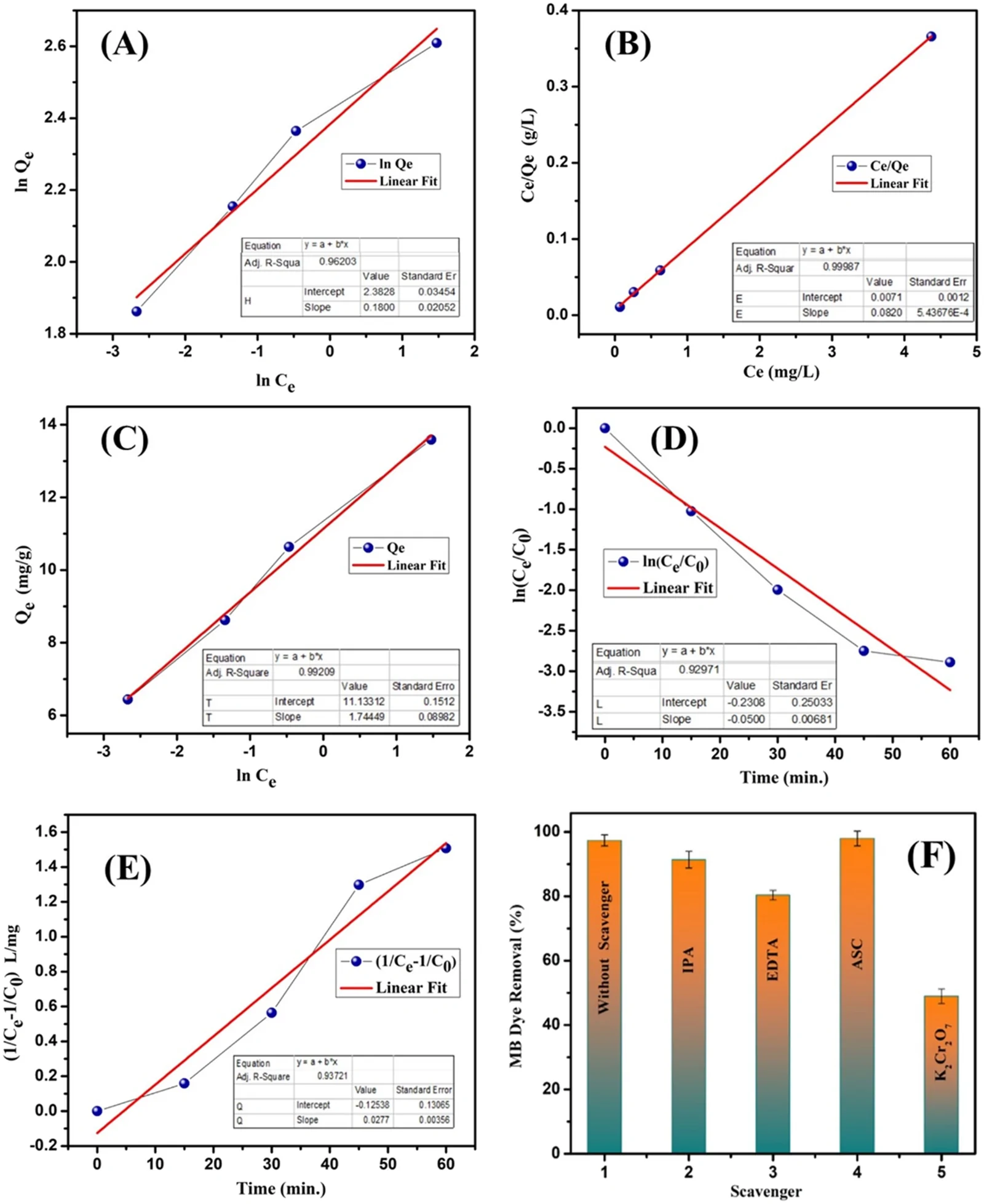
Linear isotherm plots of (A) Freundlich, (B) Langmuir and (C) Temkin models (for *C*_0_ = 10–25 ppm, NFs = 0.02 g ml^−1^); linear kinetic fits for (D) pseudo-first order and (E) pseudo-second order studies; and (F) active species trapping experiment for adsorption mechanism studies of MB dye (*C*_0_ = 20 ppm), on (0.02 g ml^−1^) CuO–ZrO_2_@PAN NFs.

The removal of MB dye by the synthesised CuO–ZrO_2_@PAN nanofibers was evaluated using the Freundlich, Langmuir and Temkin isotherm models, along with linear pseudo-first and second order kinetic models to elucidate the nature and efficiency of the MB dye removal process (Fig. 5(A)–(E)). Among the isotherm models, the Langmuir model exhibited the best linear fit with an *R*^2^ value of 0.9998, suggesting that MB dye molecules form a monolayer on a homogeneous adsorbent surface, suggesting efficient electron transfer and charge separation at active sites, where no further adsorption occurs once a site is occupied.^53^ This indicated a strong specific interaction between the dye molecules and the catalyst surface. In contrast, the Freundlich isotherm, with an *R*^2^ of 0.962, suggested a heterogeneous adsorption surface with sites of varying affinities and possible multilayer adsorption.^54^ The Temkin isotherm (*R*^2^ = 0.992) implied that adsorption heat decreases linearly with surface coverage due to the MB molecules and catalytic surface interactions.^55^ The high regression value for Temkin also supports the presence of moderate interactions and includes surface heterogeneity on nanofibers (Table 2). For kinetic modelling, the pseudo-second-order model achieved a higher *R*^2^ (0.937) compared to the pseudo-first-order model (*R*^2^ = 0.929), confirming that chemisorption is the dominant rate-limiting mechanism, likely involving electron sharing or exchange between nanofiber surface sites and MB dye molecules. These results collectively indicated that the removal process of MB dye was best explained by the Langmuir isotherm and pseudo-second-order kinetics, confirming that the process involved monolayer chemisorption with high affinity and efficient utilization of active adsorption sites. For the key mechanistic insight of the photocatalytic MB dye removal, specific scavengers were employed (Fig. 5(F)) to identify the predominant reactive species involved in the degradation process.^15^ The marked decrease in the MB removal efficiency % upon addition of potassium dichromate (K_2_Cr_2_O_7_), a known electron scavenger, indicates that photogenerated electrons (e^−^) play a crucial role in the degradation mechanism. On the other hand, the presence of isopropanol (IPA) and ethylenediaminetetraacetic acid (EDTA), which are hydroxyl radical (˙OH) and hole (h^+^) scavengers, respectively, also resulted in moderate suppression of activity, suggesting that both ˙OH and h^+^ contribute to the process, but as secondary species. The negligible impact of ascorbic acid (ASC), a superoxide radical (˙O_2_^−^) scavenger, implies that they (˙O_2_^−^) are not significantly involved in the MB dye degradation pathways in this system. These observations hence highlight and possibly suggest a mechanism primarily driven by the direct electron transfer and subsequent redox reactions involving holes (h^+^) and hydroxyl radicals (˙OH), reinforcing the importance of efficient charge separation and electron participation at the photocatalyst surface due to the PAN matrix as a proposed reason for the removal of the dye.^56^

**Table 2:** Isotherm model parameter comparison

Isotherm model	Parameters	Values
Freundlich	*K*_F_ (L g^−1^)	10.83
	1/*n*	0.1800
	*R* ^2^	0.9620
Langmuir	*Q*_m_ (mg g^−1^)	12.20
	*K*_L_ (L mg^−1^)	11.55
	*R* ^2^	0.9998
Temkin	*B* (J mol^−1^)	1.745
	*K*_T_	593.77
	*R* ^2^	0.992

### 3.4. Mechanism

Upon UV-C irradiation (*λ* = 254 nm), the CuO–ZrO_2_@PAN nanofibers might have exhibited photocatalytic activity through a synergistic mechanism involving interfacial charge transfer, redox cycling and reactive oxygen species (ROS) generation.^57^ The Type-II band alignment between CuO and ZrO_2_ may arise here due to the possibility of the staggered alignment of their conduction and valence bands as reported in other works^58–60^ (Fig. S6), where the conduction band (CB) of ZrO_2_ is at a higher energy level than that of CuO, and the valence band (VB) of CuO lies above that of ZrO_2_.^16,59^ This might have facilitated the migration of photogenerated electrons (e^−^) from the CB of ZrO_2_ to the CB of CuO, while holes (h^+^) accumulate in the VB of ZrO_2_, enhancing charge separation and possibly reducing recombination rates. The thermal treatment of nanofibers stabilised polyacrylonitrile (PAN), inducing partial cyclization, forming N-rich conjugate structures that served as electron reservoirs and a physical matrix for metal oxides.^61^ These domains might have suppressed electron–hole recombination and improved the overall stability of the photocatalyst.

The possible steps for the proposed mechanism are given below:

(i) Photon absorption and carrier generationCuO–ZrO_2_@PAN + *hν* → e^−^_CB_ + h^+^_VB_

(ii) Charge transfer (type-II heterojunction behaviour)e^−^_CB(ZrO_2_)_ → e^−^_CB(CuO)_h^+^_VB(CuO)_ → h^+^_VB(ZrO_2_)_

(iii) Reduction of dissolved oxygenO_2_ + e^+^ → ˙O_2_^−^˙O_2_^−^ + 2H^+^ + e^−^ → H_2_O_2_H_2_O_2_ + e^−^ → ˙OH + OH^−^

(iv) Cu(ii)/Cu(i) redox cycleCu^2+^ + e^−^ → Cu^+^Cu^+^ + H_2_O_2_ → Cu^2+^ + ˙OH + OH^−^

(v) Oxidation of water or hydroxide ions by photogenerated holesH_2_O + h^+^ → ˙OH + H^+^OH^−^ + h^+^ → ˙OH

(vi) Degradation of Methylene Blue (MB) by the radicalsMB dye + ˙OH → CO_2_ + H_2_O

### 3.5. Other dyes

**Fig. 6:**
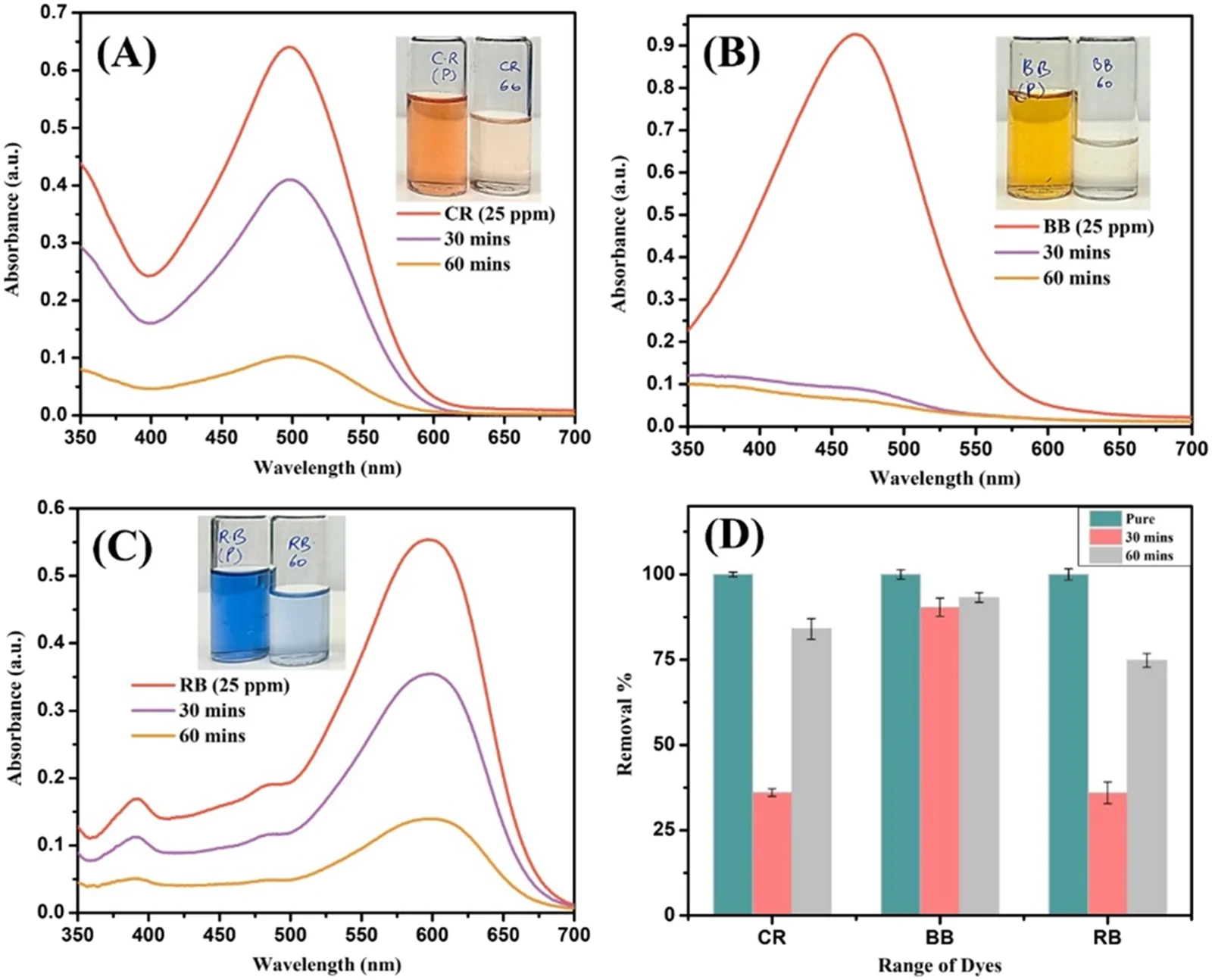
Degradation performance of the photocatalyst NFs on other model dyes: (A) Congo Red (CR), (B) Bismarck Brown (BB), and (C) Reactive Black (RB) and (D) comparison of removal studies.

Apart from MB dye degradation, the prepared catalyst was also primarily tested for other model dyes, namely Congo Red (CR), Bismarck Brown (BB) and Reactive Black (RB), to evaluate its applicability in degrading a broader range of textile dye effluents. The photocatalytic degradation performance was monitored *via* UV-vis absorption spectroscopy at intervals of 0, 30 and 60 minutes, as shown in Fig. 6(A)–(C), with the corresponding dye removal percentages given in Fig. 6(D).

The dyes were also tested for degradation under UV light alone for photolysis as well (Fig. S1). For Congo Red (CR), the initial spectrum exhibited a prominent absorption peak near 497 nm. Upon visible-light irradiation in the presence of the CuO–ZrO_2_@PAN catalyst, the peak intensity decreased significantly within 30 minutes and disappeared by 60 minutes, indicating a degradation efficiency of nearly 85%. This high performance is likely due to strong dye adsorption and effective photoinduced charge separation at the CuO–ZrO_2_ interface supported on the PAN matrix.^62,63^ A sharp increase in the absorbance was recorded with increasing irradiation time for Bismarck Brown (BB) with an initial peak near 460 nm, resulting in over 90% removal after 60 minutes. The catalyst's ability to degrade this azo dye suggests excellent reactivity towards the complex aromatic dye structure, often resistant to conventional treatments.^63^ Reactive Black (RB), on the other hand, showed a more gradual degradation. The peak around 554 nm declined slowly, achieving approximately 75% removal after 60 minutes.

The comparatively lower efficiency may be attributed to the steric hindrance/complexity and stability of the anionic^64^ RB dye molecules, which could possibly limit their interaction with the catalyst's active surface sites. The overall degradation of the dyes was then compared (Fig. 6(D)) at 30 min and then at the end of 60 min. This highlighted the capability of the fabricated photoactive filter (PAF) for the removal of a broad range of both cationic and anionic dyes as compared to the other reported works (Table S1).

### 3.6. Ecotoxicological assay

**Fig. 7:**
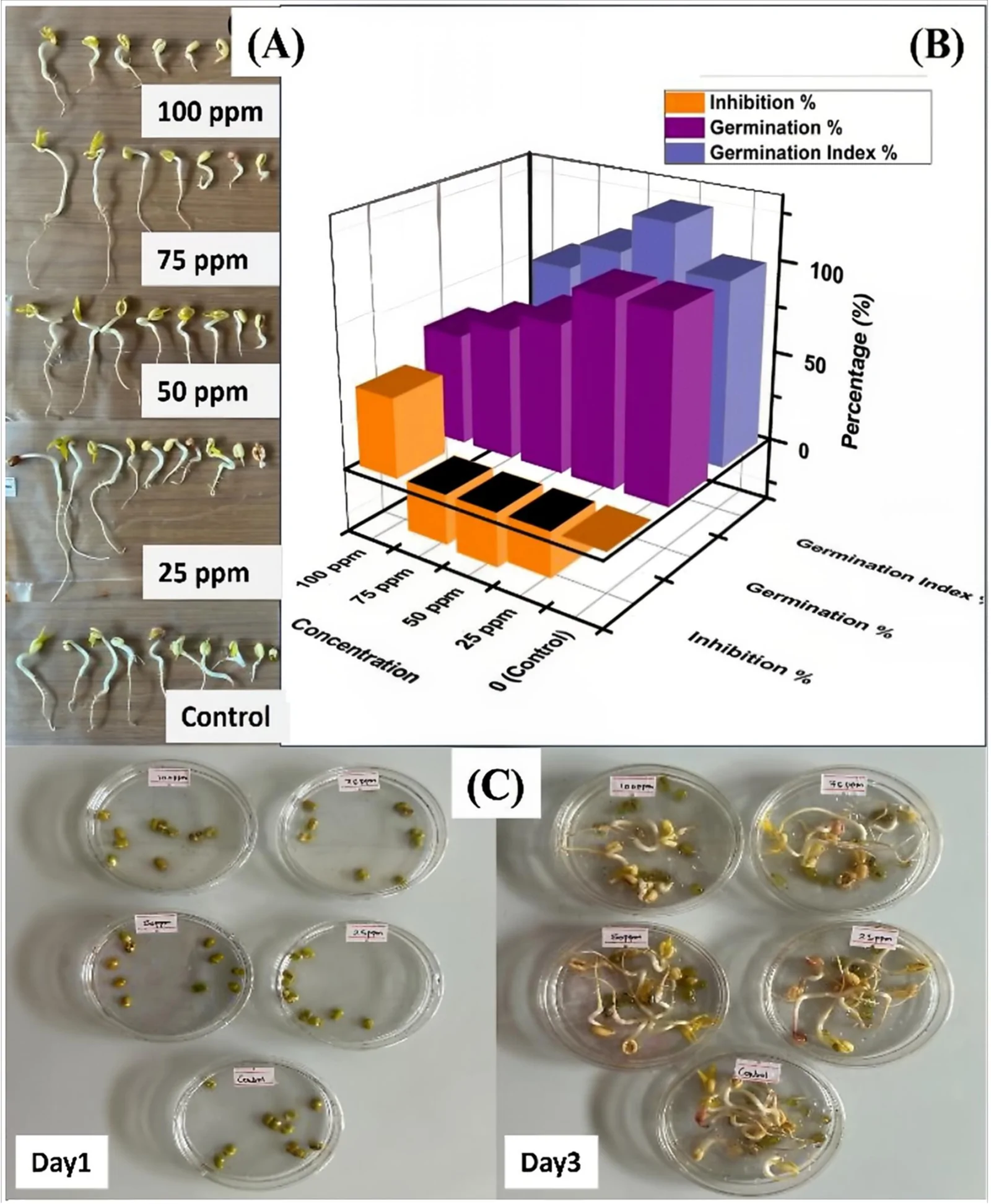
Effects of different concentrations of CuO–ZrO_2_@PAN NFs on (A) the root length and (B) % inhibition, germination and germination index measurements for *Vigna radiata* seeds and (C) the visual image of the germination of the seeds from day 1 to day 3.

The eco-toxicological impact of CuO–ZrO_2_@PAN nanofibers on *Vigna radiata* (green gram) seed germination and early root development was assessed over a period of 72 h in an incubation chamber at room temperature and under dark conditions to mimic the conditions in soil. The results indicated a concentration-dependent response. At lower concentrations (25, 50, and 75 ppm), root elongation was stimulated compared to the control, suggesting a hormetic effect.^65,66^ Specifically, the highest mean root length (6.175 cm) was observed at 50 ppm (Fig. 7(A)), corresponding to a −29.19% inhibition value (Table 3), where the negative value indicates the reverse of inhibition, *i.e.* positive growth with respect to the control.

**Table 3:** Seed germination measurements

Concentration (ppm)	Germination %	Mean root length (cm)	Germination index %	Inhibition %
0 (control)	100	4.78	4.78	0
25	100	5.94	5.94	−24.27
50	80	6.175	4.94	−26.99
75	70	6.07	4.25	−29.19
100	60	2.7	1.62	43.51

Conversely, at 100 ppm, a significant reduction in root length (2.7 cm) and germination percentage (60%) was noted, indicating slight phytotoxicity.

This trend implies that the nanofibers may act as a micronutrient supplement at sub-toxic/controlled concentrations, consistent with the previously reported hermetic dose responses in nanotoxicology.^65^ The germination index followed a similar pattern, with the maximum value at 25 ppm and a sharp decline at 100 ppm (Fig. 7(B)). The visible growth of the seeds from day 1 to day 3 can be observed in Fig. 7(C).

These findings highlight the importance of dose in determining nanoparticle bioactivity and toxicity in plant systems. Hence, the CuO–ZrO_2_@PAN nanofibers are safe for the environment up to quite high concentrations before they inhibit plant growth.

## 4. Conclusions

In this study, novel CuO–ZrO_2_@PAN photoactive filter/nanofibers were successfully synthesised and characterized for their efficacy in the degradation of toxic dyes from aqueous media. The catalyst demonstrated remarkable potential in the photocatalytic degradation of multiple dyes, including MB dye, achieving a degradation efficiency of ∼98% within 60 minutes of contact time under optimal conditions (*C*_0_ = 20 ppm, 0.02 g ml^−1^ of 40 wt% CuO–ZrO_2_@PAN NFs at RT).

These are also the first reported bimetal oxide-based nanofibers to successfully exhibit high photocatalytic degradation efficiency against structurally diverse azo and thiazine dyes at concentrations up to 25 ppm. For instance, apart from MB dye, the removal efficiencies for three other tested dyes within 60 minutes were 90% for BB, 85% for CR and 75% for RB dye. These results highlighted the material's broad-spectrum capability, rapid kinetics, and pioneering potential as a multifunctional nanofiber-based photocatalytic platform for dye removal in wastewater treatment that has not been reported yet. The fibrous morphology of the photoactive filter/nanofibers provided structural stability, while the embedded CuO–ZrO_2_ nanoparticles increased the active surface sites, crucial for adsorption and catalytic degradation of pollutants. Overall, the TEM and SEM analysis confirmed the formation of a fibrous PAN matrix with fibres of ∼291 nm embedded with CuO–ZrO_2_ NPs (ranging from ∼10 to 30 nm), allowing for excellent dispersion of active sites, and well-defined crystallinity. Kinetic models and isotherms also indicated a highly surface-controlled mechanism. An ecotoxicological study *via* seed germination assay highlighted high environmental safety limits for the NFs, thus making them promising materials for wastewater remediation processes. In the future, detailed band edge characterization through cyclic voltammetry (CV) and electrochemical impedance spectroscopy (EIS) will be undertaken to experimentally validate the proposed band alignment. Additionally, by-product analysis and total organic carbon (TOC) measurements will be performed to evaluate the extent of mineralization to further confirm the environmental safety of the photocatalytic process. Furthermore, studies will also explore alternative metal oxide combinations and polymeric supports to design enhanced broad spectrum and environmentally safe photocatalytic filter materials.

## Author contributions

Conceptualization, C. R. C. and K. R.; methodology, K. R., J. V. C., and C. R. C.; resources, C. R. C. and A. J. S.; visualization, K. R. and C. R. C.; writing – original draft, K. R. and C. R. C.; writing – review & editing, C. R. C., J. V. C., A. J. S., and M. D. S.; project administration, C. C., A. J. S., and M. D. S.; funding acquisition, C. R. C., A. J. S. and K. R.; and supervision, C. R. C., A. J. S., and M. D. S.

## Conflicts of interest

There are no conflicts to declare.

## Supplementary Material

LP-004-D5LP00307E-s001

## Data Availability

The data supporting this article have been included as part of the supplementary information (SI). Supplementary information is available. See DOI: https://doi.org/10.1039/d5lp00307e.
