# Supplementary material for: Recyclable and eco-compatible polymer composite nanofibers for broad-spectrum dye remediation
Source: RSC Appl Polym. 2026 Jan 19;4(2):725–35. doi: 10.1039/d5lp00307e (PMC13592383; doi:10.1039/d5lp00307e)
Supplement: Supplementary file 1 [file LP-004-D5LP00307E-s001.pdf]

Supplementary Information (SI) for RSC Applied Polymers.
This journal is © The Royal Society of Chemistry 2026

Supplementary Data

Figure S1: Effect of direct UV light irradiation alone on (A) MB (B) CR (C) BB and (D) RB dyes for 60 minutes each.

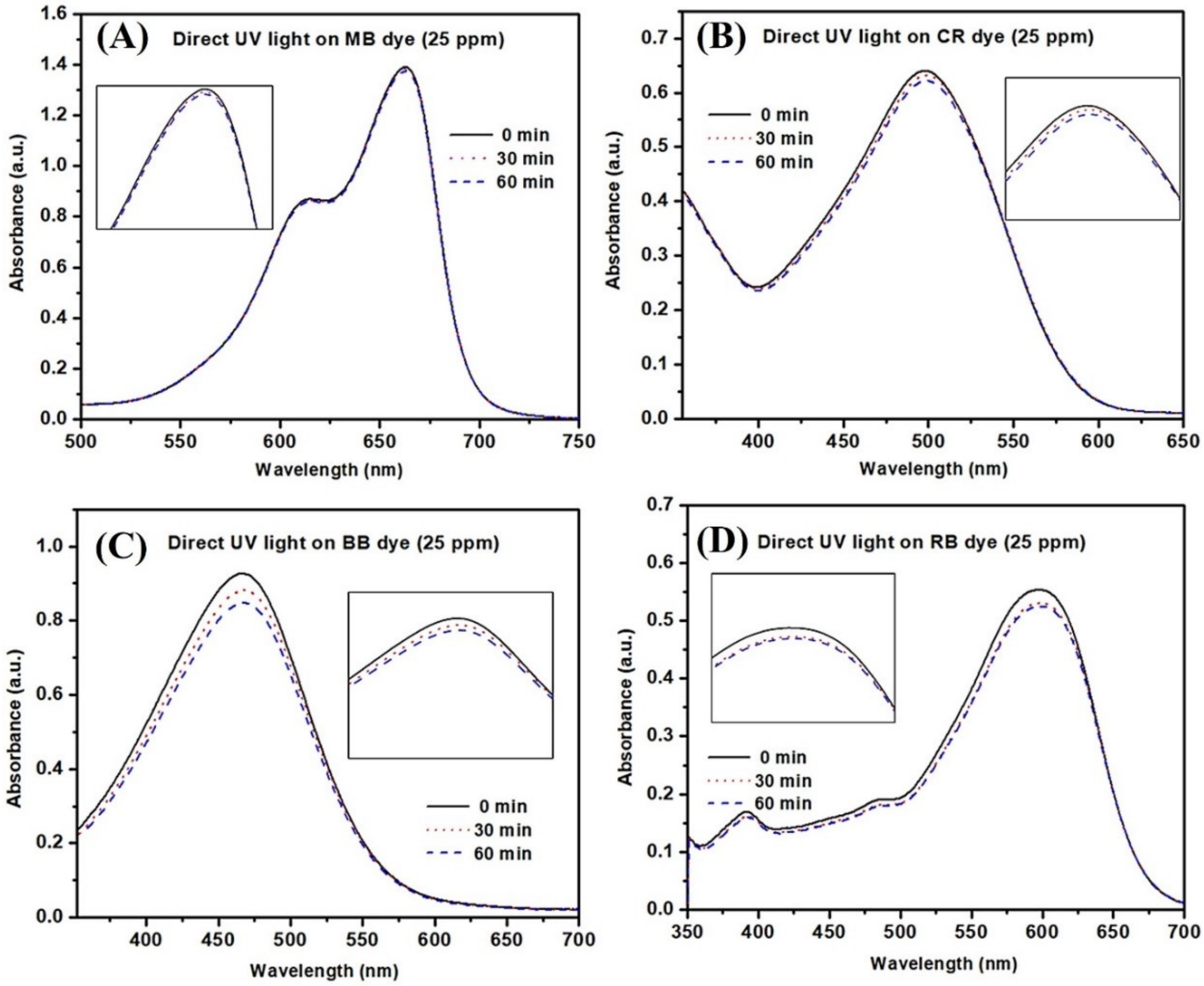

Figure S2: TGA studies of the CuO-ZrO_2@PAN fibers at 40 wt.%

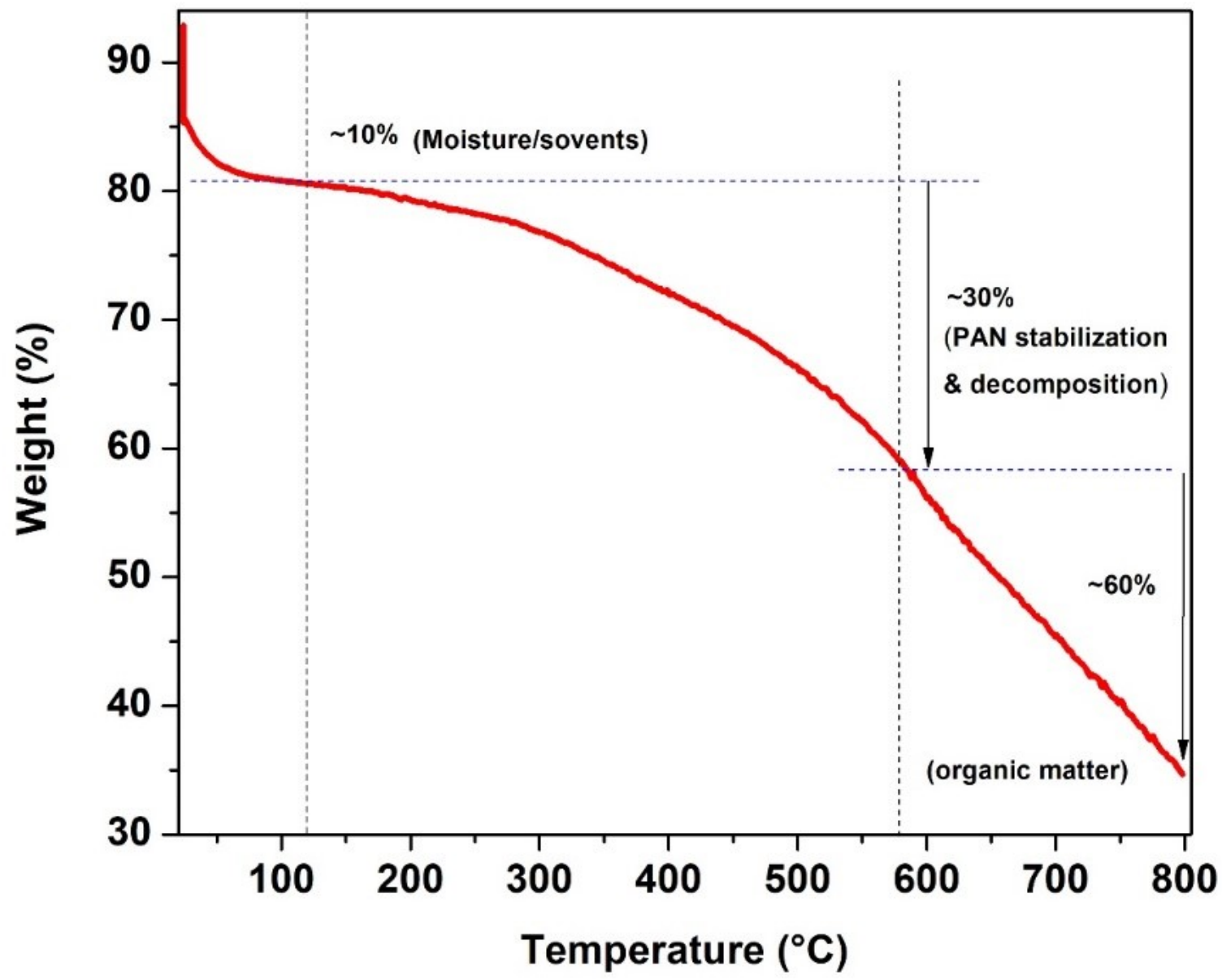

Figure S3: Roughness of Synthesised Fibers is clearly visible

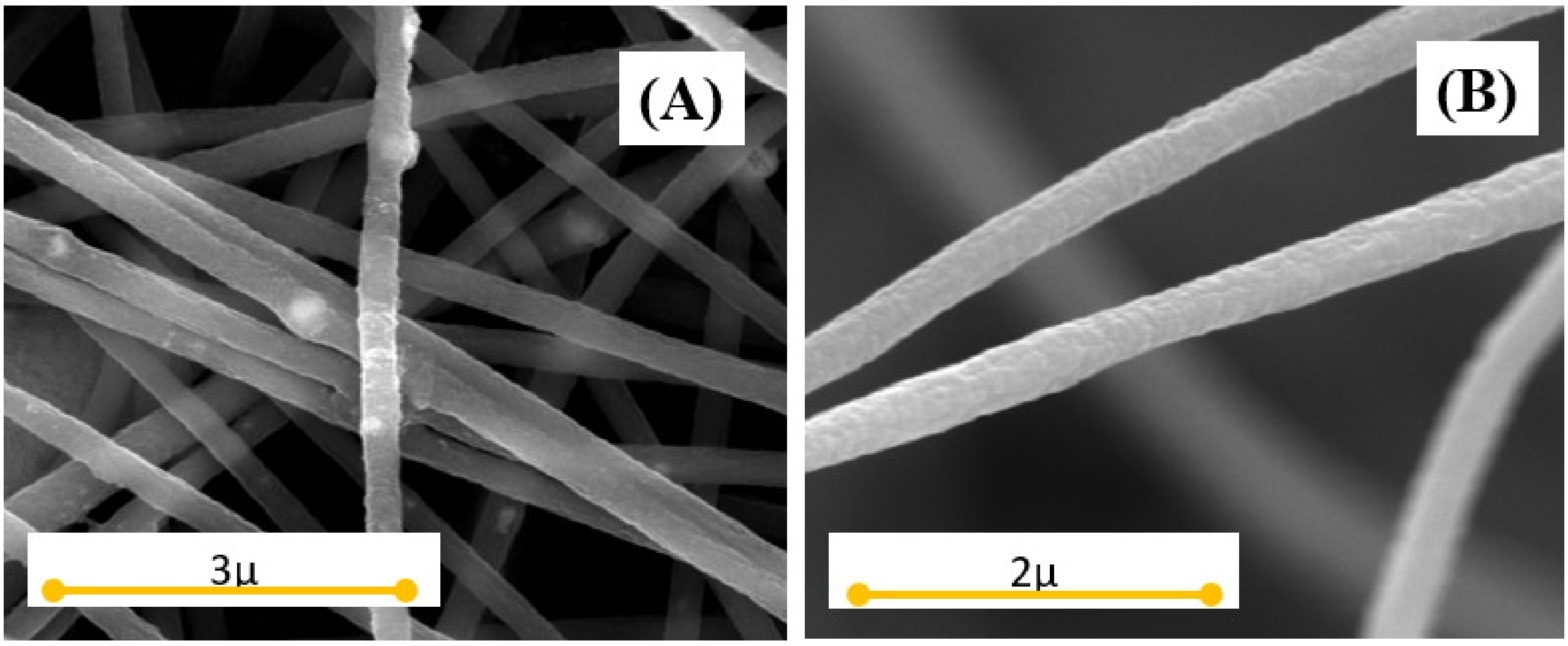

Figure S4: **(A)**The average size of the CuO-ZrO_2@PAN Nanofibers NPs using ImageJ **(B)** Band gap energy of the fibers **(C)** TEM image of CuO-ZrO_2 NPs **(D)** average size of the CuO-ZrO_2 NPs

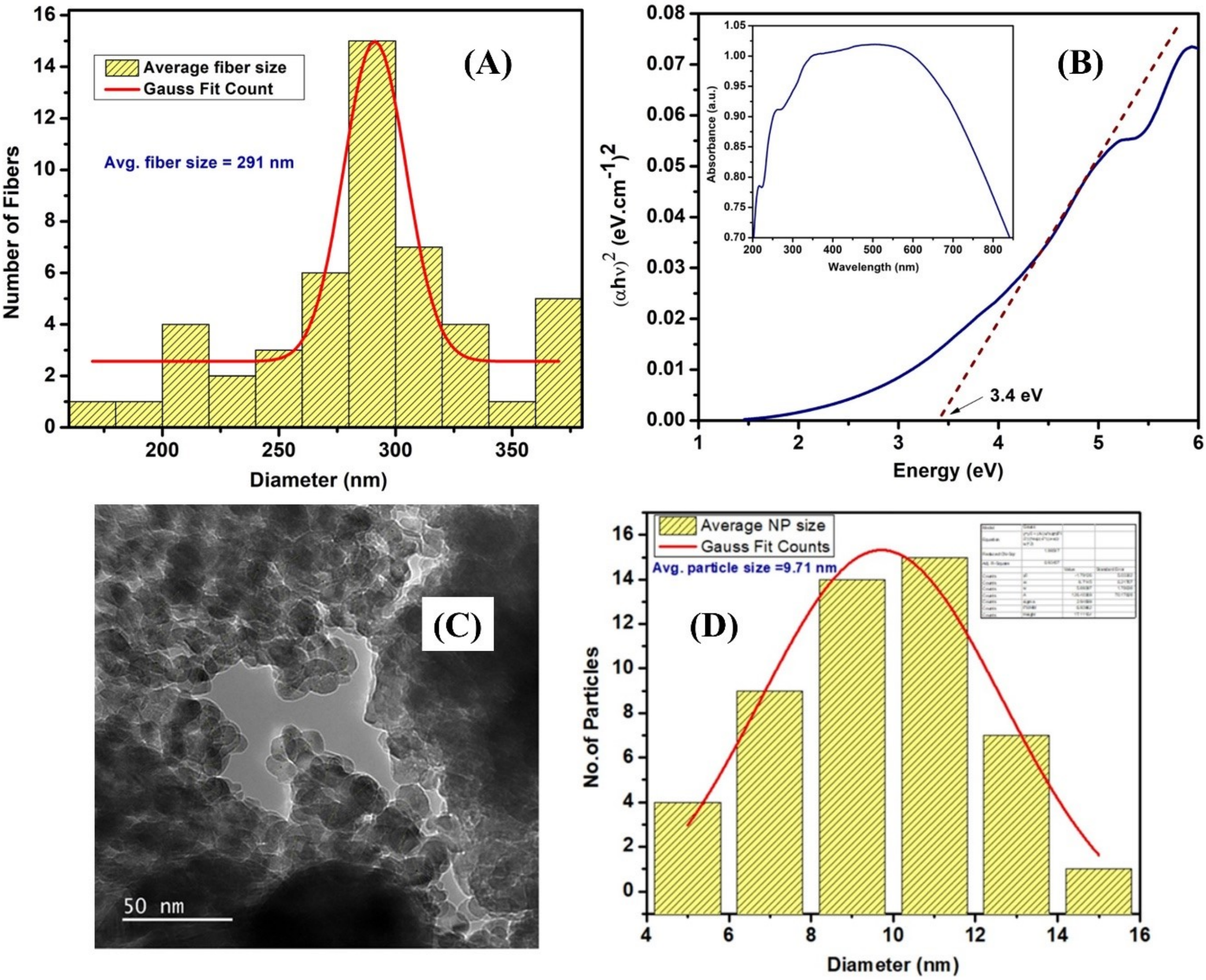

Figure S5: (A) The dark adsorption results for 30 minutes with different composition % and **(B)** Recyclability studies for five consecutive cycles for MB dye

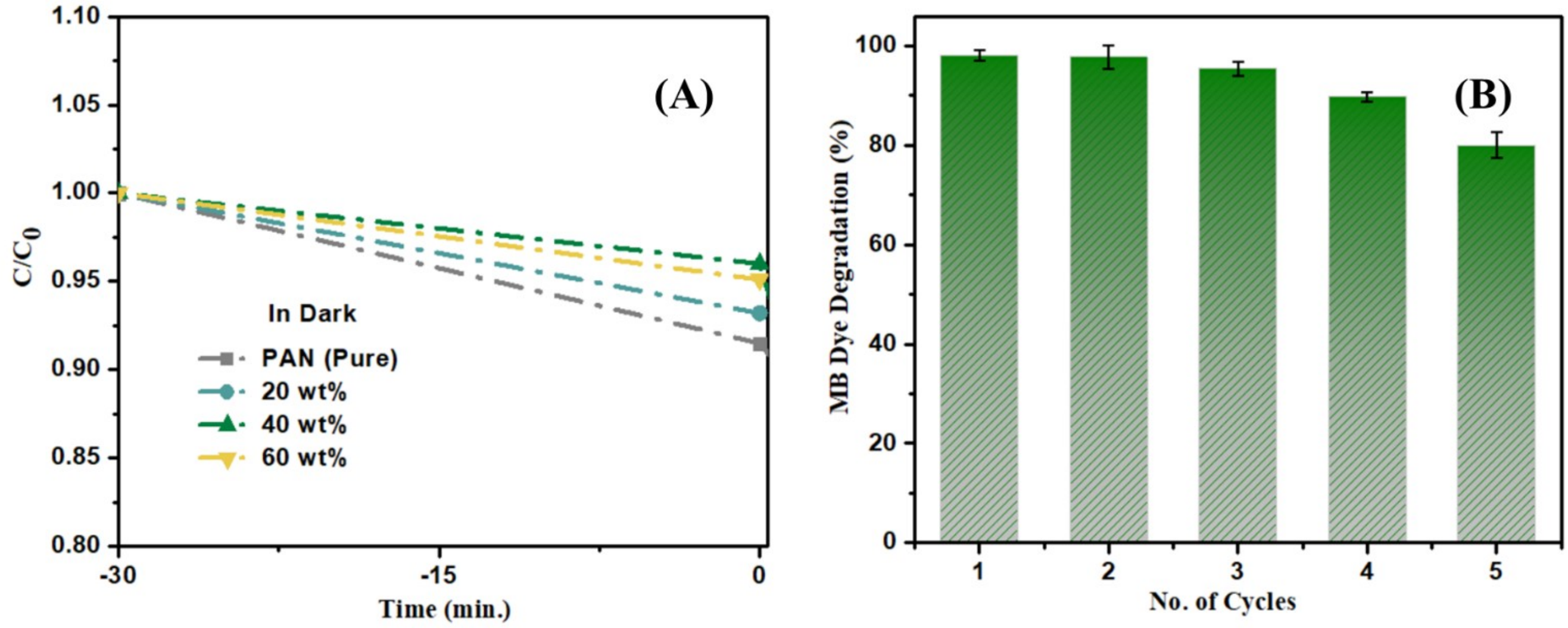

Figure S6: The Band gap suggested of both CuO, ZrO_2 and CuO-ZrO_2 NPs to understand the Type II alignment

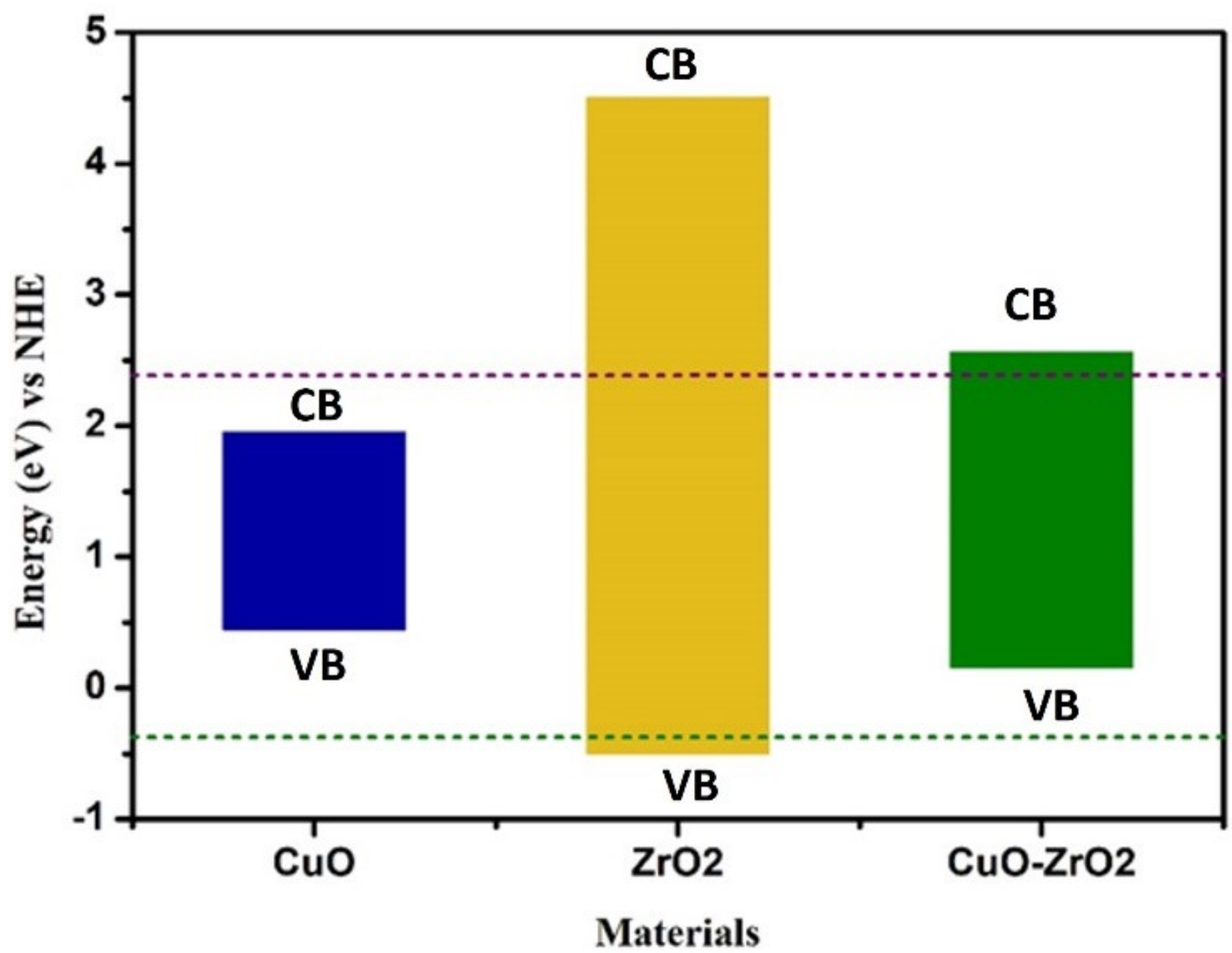

Table S1: Performance Comparison of previously reported materials and presented work of CuO-ZrO_2@PAN NFs

Material Used	No. Of Dyes targeted	Degradation %	Nanosystem type	Time Taken	Reference s

5% Ta-doped TNF	1	MB	Metal-metal oxide Nanofibers	>200 min	[1]
Ag_2O/ZnO-TiO_2	1	RhB (94%)	Tertiary nanocomposite	150 min	[2]
Cu_2O-CuO/TiO_2 on Cu wires	2	MB, MO (87%)	Bi metal oxide nanorodes	120 min	[3]
TiO_2-GO	2	MG (85%), MB (93%)	Nanocomposite	60 min	[4]
Zr/Ag co-doped TiO_2 NFs	1	CR (99.3%)	Tertiary Nanofiber	120 min	[5]
CeO_2 NFs	1	MB (97.6%)	Metal oxide Nanofibers	180 min	[6]
Ag-ZnO NFs	1	RhB	Metal-metal oxide Nanofibers	50 min	[7]
Fe@TiSi	1	MB (86.8%)	Tertiary core shell nanofibers	60 min	[8]
Ag-H:TiO_2 NFs	1	Brilliant Green (BG)	hydrogenated TiO_2 nanofibers decorated metal nanoparticles	100-150 min	[9]
AuAg-mTNF-H	1	MB	Mesoporous nanofibers with alloy nanoparticles	90 min	[10]
CuO-ZrO_2@PAN	4	MB (98%)<br>BB (90%)<br>CR (85%)<br>RB (~75%)	Bi-metal oxide in electro spun Nanofibers	60 min	**Present work**

References

[1] N. Singh, J. Prakash, M. Misra, A. Sharma, and R. K. Gupta, *ACS applied materials and interfaces*, 2017, **9(34)**, 28495-28507.

[2] S. I. Wani and A. S. Ganie, *Inorganic Chemistry Communications*, 2021,**128**, 108567.

[3] F. Ansari and S. Sheibani, *Journal of Alloys and Compounds*, 2022, **919**.

[4] N. Verma, T. S. Chundawat, H. Chandra, and D. Vaya, *Materials Research Bulletin*, 2023, **158**, 112043.

[5] W. Qi, Y. Yang, J. Du, J. Yang, L. Guo, and L. Zhao, *Journal of Colloid and Interface Science,* 2021, **603**, 594–603.

[6] L. Zhu, H. Li, P. Xia, Z. Liu, and D. Xiong, *ACS applied materials and interfaces*, 2018, **10(46)**, 2-10.

[7] D. Lin, H. Wu, R. Zhang, and W. Pan, *Chemistry of Materials* ,2009, **21(15)**, 3479-3484.

[8] S. Zhan, D.Zhu, G.Ren, Z. Shen, M. Qui, S.Yang, H.Yu, Y.Li, *ACS applied materials and interfaces*, 2014, **6(19)**, 16841-16850.

[9] M.C Wu,W.K. Huang,T.H. Lin, Y.J. Lu, *Applied Surface Science,* 2019, **469**, 34-43.

[10] S. Chattopadhyay, S. Bysakh, P. M. Mishra, and G. De, *Langmuir*, 2019, **35(44)**, 14364-14375.
